# Preventive Effect of Dipeptidyl Peptidase-4 Inhibitor on Atherosclerosis Is Mainly Attributable to Incretin's Actions in Nondiabetic and Diabetic Apolipoprotein E-Null Mice

**DOI:** 10.1371/journal.pone.0070933

**Published:** 2013-08-13

**Authors:** Michishige Terasaki, Masaharu Nagashima, Kyoko Nohtomi, Kyoko Kohashi, Masako Tomoyasu, Kyoko Sinmura, Yukinori Nogi, Yuki Katayama, Kengo Sato, Fumiko Itoh, Takuya Watanabe, Tsutomu Hirano

**Affiliations:** 1 Department of Medicine, Division of Diabetes, Metabolism, and Endocrinology, Showa University School of Medicine, Shinagawa-ku, Tokyo, Japan; 2 Laboratory of Cardiovascular Medicine, Tokyo University of Pharmacy and Life Sciences, Hachioji-City, Tokyo, Japan; University of Leicester, United Kingdom

## Abstract

**Aim:**

Several recent reports have revealed that dipeptidyl peptidase (DPP)-4 inhibitors have suppressive effects on atherosclerosis in apolipoprotein E-null (*Apoe* ^−/−^) mice. It remains to be seen, however, whether this effect stems from increased levels of the two active incretins, glucagon-like peptide-1 (GLP-1) and glucose-dependent insulinotropic polypeptide (GIP).

**Methods:**

Nontreated *Apoe* ^−/−^ mice, streptozotocin-induced diabetic *Apoe* ^−/−^ mice, and *db/db* diabetic mice were administered the DPP-4 inhibitor vildagliptin in drinking water and co-infused with either saline, the GLP-1 receptor blocker, exendin(9–39), the GIP receptor blocker, (Pro^3^)GIP, or both *via* osmotic minipumps for 4 weeks. Aortic atherosclerosis and oxidized low-density lipoprotein-induced foam cell formation in exudate peritoneal macrophages were determined.

**Results:**

Vildagliptin increased plasma GLP-1 and GIP levels without affecting food intake, body weight, blood pressure, or plasma lipid profile in any of the animals tested, though it reduced HbA1c in the diabetic mice. Diabetic *Apoe* ^−/−^ mice exhibited further-progressed atherosclerotic lesions and foam cell formation compared with nondiabetic counterparts. Nondiabetic and diabetic *Apoe* ^−/−^ mice showed a comparable response to vildagliptin, namely, remarkable suppression of atherosclerotic lesions with macrophage accumulation and foam cell formation in peritoneal macrophages. Exendin(9–39) or (Pro^3^)GIP partially attenuated the vildagliptin-induced suppression of atherosclerosis. The two blockers in combination abolished the anti-atherosclerotic effect of vildagliptin in nondiabetic mice but only partly attenuated it in diabetic mice. Vildagliptin suppressed macrophage foam cell formation in nondiabetic and diabetic mice, and this suppressive effect was abolished by infusions with exendin(9–39)+(Pro^3^)GIP. Incubation of DPP-4 or vildagliptin *in vitro* had no effect on macrophage foam cell formation.

**Conclusions:**

Vildagliptin confers a substantial anti-atherosclerotic effect in both nondiabetic and diabetic mice, mainly *via* the action of the two incretins. However, the partial attenuation of atherosclerotic lesions by the dual incretin receptor antagonists in diabetic mice implies that vildagliptin confers a partial anti-atherogenic effect beyond that from the incretins.

## Introduction

Incretin-based therapies have been reported to suppress the development of atherosclerosis and its related diseases by ameliorating hyperglycemia, decreasing blood pressure and atherogenic lipoproteins, and improving vascular inflammation and endothelial dysfunction [1], [2], [3]. Our group previously reported that the subcutaneous infusion of human native glucagon-like peptide-1 (GLP-1) suppressed the development of atherosclerotic lesions in apolipoprotein E-null (*Apoe* ^−/−^) mice, a representative animal model of atherosclerosis [4]. Surprisingly, we also found that the infusion of human native glucose-dependent insulinotropic polypeptide (GIP) suppressed the development of atherosclerotic lesions in *Apoe* ^−/−^ mice as potently as GLP-1 infusion in the same animal model [4]. If GLP-1 and GIP both have anti-atherogenic properties, the increases of both active GLP-1 and active GIP by dipeptidyl peptidase (DPP)-4 inhibitor treatment are likely to suppress the development of atherosclerotic lesions in an additive fashion. Several recent reports, including our own, have actually shown that DPP-4 inhibitors significantly suppress the development of atherosclerotic lesions in animal models [5]–[9]. It remains unknown, however, whether this anti-atherogenic property of DPP-4 inhibitor can be credited to higher endogenous levels of the active incretins GLP-1 and GIP. Much attention has been drawn to the new hypothesis that DPP-4 inhibitors confer anti-atherogenic effects by blocking proinflammatory and proatherogenic properties of DPP-4 that is also known as CD26 (DPP-4/CD26) [3], [5], [6], [9], [10]. If the anti-atherosclerotic effect of DPP-4 inhibitor is derived from DPP-4 inactivation itself, DPP-4 inhibitor can elicit anti-atherogenic effects even when no increases in the active incretins take place.

**Figure 1:**
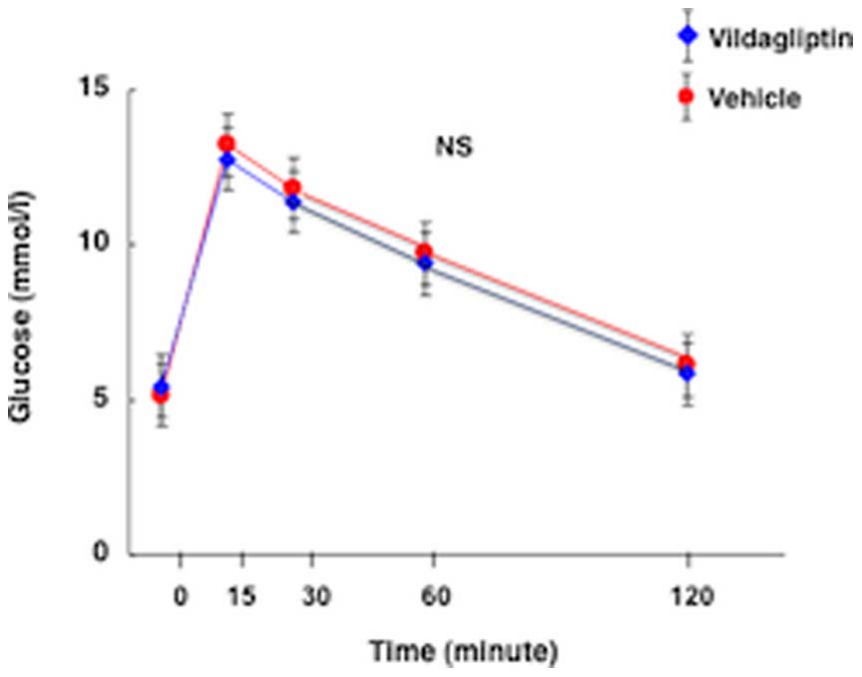
Oral glucose tolerance test (1.5 mg/g body weight) in nondiabetic *Apoe* ^−**/**−^ mice without (vehicle) and with vildagliptin treatment.

In the present study we attempted to determine whether anti-atherogenic property of DPP-4 inhibitor derives from increased levels of the endogenous active incretins GLP-1 and GIP, or from other mechanisms such as DPP-4 inactivation. To achieve this, we administered GLP-1 and GIP receptor antagonists to *Apoe* ^−/−^ mice simultaneously treated with the DPP-4 inhibitor vildagliptin and examined how the incretin receptor blockade attenuated the DPP-4 inhibitor's anti-atherosclerotic effect.

The GIP receptors (GIPRs) are severely down-regulated in diabetes [11], [12], and the incretin effect of GIP is virtually abolished in diabetic subjects [13]. It thus seems unlikely that GIP can exert its anti-atherogenic property in diabetes. In a recent study by our group, however, GIP infusion suppressed the development of atherosclerosis in streptozotocin (STZ)-induced *Apoe* ^−/−^ mice [14]. In the present study we tried to determine whether the endogenous elevation of active incretins in diabetic *Apoe* ^−/−^ mice by DPP-4 inhibition exerts an anti-atherogenic effect comparable to that seen in nondiabetic *Apoe* ^−/−^ mice.

## Materials and Methods

### Animal experiments

This study was carried out in strict accordance with the recommendations in the Guide for the Care and Use of Laboratory Animals of the National Institutes of Health. The protocol was approved by the Institutional Animal Care and Use Committee of Showa University (Permit Number: 02070). All surgery was performed under diethyl ether anesthesia, and all efforts were made to minimize suffering.

#### Experiment 1

One hundred and sixty 8-week-old male *Apoe* ^−/−^ mice were purchased from Sankyo Labo Service (Tokyo, Japan) and kept on a normal chow for 9 weeks. Upon reaching 17 weeks of age, they were switched to an atherogenic diet containing 30% fat, 20% sucrose, 8% NaCl, and 0.15% cholesterol (Oriental Yeast, Tokyo, Japan) [4], [14]. A DPP-4 inhibitor, vildagliptin, was kindly provided by Novartis Pharma (Basel, Switzerland). Starting from the same point, 17 weeks of age, vildagliptin was administered in drinking water for 4 weeks at 0.003% w/v, a concentration determined to deliver 3 mg/kg/day in normal mice [15]. Over this 4-week period of vildagliptin administration, the *Apoe* ^−/−^ mice were respectively infused with GLP-1 receptor (R) blocker, exendin(9–39) (Ex-9, 22 nmol/kg/day, AnaSpec, San Jose, CA), the GIPR blocker, (Pro^3^)GIP (Pro^3^, 25 nmol/kg/day, Abgent, San Diego, CA), Ex-9+Pro^3^, or saline (vehicle) for 4 weeks by osmotic mini-pump (Alzet Model 1007D, Durect, Cupertino, CA) [16]. A subset of 17-week-old *Apoe* ^−/−^ mice without vildagliptin were infused with Ex-9+Pro^3^ for 4 weeks.

#### Experiment 2

When the *Apoe* ^−/−^ mice reached the age of 15 weeks, 90 animals were given multiple peritoneal injections of STZ (50 mg/kg/day) for 5 consecutive days to induce diabetes, according to the method described by Takeda *et al.* [17]. Eight mice died of STZ-induced toxicity. Two weeks later, all of the remaining 82 mice with a 6-hour fasting blood glucose (Glutest Sensor, Sanwa Chemical, Tokyo, Japan) of greater than 11 mmol/l were selected as the diabetic group. Starting from 17 weeks of age, the diabetic *Apoe* ^−/−^ mice were given drinking water with or without vildagliptin (0.003% w/v) and fed an atherogenic diet for 4 weeks. The 17-week-old diabetic *Apoe* ^−/−^ mice were respectively infused with Ex-9, Pro^3^, Ex-9+ Pro^3^, and saline for 4 weeks by osmotic mini-pump in a fashion similar to that described for the nondiabetic mice.

#### Experiment 3

Thirty three *db/db* mice, a mouse model of type 2 diabetes, were purchased from Sankyo Labo Service at the age of 6 weeks and kept on normal chow. From the age of 8 weeks, a point at which diabetes is established to be active in *db/db* mice. Starting from the age of 9 weeks, the diabetic *db/db* mice were given drinking water with or without vildagliptin (0.003% w/v) for 4 weeks, and those that received the vildagliptin were infused with Ex-9 (22 nmol/kg/day)+Pro^3^ (25 nmol/kg/day).

### Measurements

After the 4 weeks of vildagliptin administration with or without infusions of incretin receptor blockers, the systolic blood pressure (SBP) and pulse rate were measured using indirect tail-cuff equipment. Blood samples were collected after a 6-hour fast. Plasma levels of glucose, total cholesterol, high-density lipoprotein (HDL) cholesterol, triglyceride, and nonesterified fatty acids (NEFA) were measured by enzymatic methods. Non-HDL cholesterol was calculated by subtracting HDL cholesterol from total cholesterol. HbA1c was measured by the quick test (A1CNow+ ® 20test-kits; Bayer Yakuhin, Osaka, Japan). Plasma levels of active GLP-1, total GLP-1, total GIP, and insulin were determined by an enzyme-linked immunosorbent assay (ELISA Kit, Millipore, MA; Ultra Sensitive “PLUS” Mouse Insulin ELISA Kit, Morinaga, Yokohama, Japan). Only total GIP was measured, as no test kit for measuring active GIP was commercially available. The plasma levels of total GIP in the Pro^3^-infused animals remained undetermined, as the test kit for total GIP was cross-reacted with Pro^3^. Oral glucose tolerance tests were performed on nondiabetic *Apoe* ^−/−^ mice with or without vildagliptin treatment after a 6-h fast. Glucose (1.5 mg/g body weight) was administered orally through a gavage tube, and blood glucose levels were measured by the glucose oxidase method using the Glucose Monitor System (Sanwa Kagaku, Nagoya, Japan).

### Atherosclerotic lesion assessment

The aorta was excised from the root to the abdominal area and the connective and adipose tissues were carefully removed. The entire aorta and cross-sections of the aortic root were stained with Oil Red O for the assessment of atherosclerotic lesions [4], [18], [19]. Macrophage infiltration into the aortic wall was visualized by anti-mouse monocyte+macrophage antibody-2 (MOMA-2) staining [4], [18], [19]. The areas of the aorta with atherosclerotic lesions were traced by an investigator blind to the treatment and measured by an image analyzer. The severity of the atheromatous lesions and degree of macrophage infiltration were assessed as sum of stained area in the aortic root [4], [18], [19].

### Cholesterol esterification assay

Exudate peritoneal cells were isolated from the treated nondiabetic or diabetic *Apoe* ^−/−^ mice at 21 weeks of age, or *db/db* diabetic mice at the age of 13 weeks, 4 days after an intraperitoneal injection of thioglycolate [18], [19]. Adherent macrophages were incubated for 18 hours with the RPMI-1640 medium containing 10 μg/ml human oxidized low-density lipoprotein (oxLDL) in the presence of 0.1 mmol/l [^3^H]oleate conjugated with bovine serum albumin. Cellular lipids were extracted and the radioactivity of the cholesterol [^3^H]oleate was determined by thin-layer chromatography [18], [19].

### Analyses of GLP-1R and GIPR expression

Peritoneal macrophages from mice and J774A.1 mouse macrophages (JCRB9108, Human Science, Osaka, Japan) were suspended in culture medium and seeded onto dishes. Aorta (vasculature), epididymal adipose tissue, pancreas, and brain were obtained from the *Apoe* ^−/−^ mice. Total RNA was extracted from the cultured cells and organs using ISOGEN reagent (Nippon-Gene, Tokyo, Japan). The cDNAs were synthesized from isolated RNA templates with a High-Capacity cDNA Archive Kit (Applied Biosystems, Carlsbad, CA). Real-time RT-PCR was performed using TaqMan Gene Expression Assays (Applied Biosystems). Pre-designed TaqMan Gene Expression system for GLP-1R and GIPR and 18S rRNA were purchased from Applied Biosystems [4], [14]. Amplification and fluorescent measurements were carried out during the elongation step with an ABI PRISM 7900 HT Sequence Detection System (Applied Biosystems). GLP-1R expression and GIPR expression were analyzed with 10 and 11 primers respectively encoding different exons of the incretin receptors (Applied Biosystems). The primers used for determination of GLP-1R and GIP gene expressions listed in the supplementary data. The amplification products visualized by gel electrophoresis had the expected lengths (bp). Intracellular concentration of cAMP in J774A.1 macrophages was measured by the ELISA kit (cAMP Assay KGE002B, R&D Systems, Minneapolis, MN).

### Analyses of DPP-4/CD26 expression

Exudate peritoneal macrophages were obtained from nondiabetic mice and STZ-induced diabetic wild-type (C57/BL) or *Apoe* ^−/−^ mice at the age of 21 weeks. DPP-4/CD26 mRNA levels were measured by real-time RT-PCR using Mm0048494538_m1 TaqMan Gene Expression Assay (Applied Biosystems).

### Effect of DPP-4/CD26 on macrophage foam cell formation

Exudate peritoneal macrophages obtained from nondiabetic *Apoe* ^−/−^ mice were incubated for 18 hours with recombinant mouse DPP-4/CD26 (100–500 ng/ml, R&D Systems) or vildagliptin (20 μmol/l) in the foam cell formation medium described above.

### Statitiscal analysis

All values are expressed as mean±SEM. Data were compared by 2-tailed unpaired Student's *t* test between 2 groups and by 1-way ANOVA followed by Bonferroni's post hoc test among 3 or more groups. Differences were considered statistically significant at *P*<0.05.

## Results

### Characteristics and laboratory data

Table 1 shows the characteristics and laboratory data from the 6 groups of *Apoe* ^−/−^ mice treated with vehicle, vildagliptin, vildagliptin with Pro^3^, vildagliptin with Ex-9, vildagliptin with Pro^3^ and Ex-9, and vehicle with Pro^3^ and Ex-9. There were virtually no differences among the groups in food intake, water intake, body weight, SBP, pulse rate, or plasma concentrations of total cholesterol, HDL cholesterol, triglyceride, NEFA, glucose, or insulin. Vildagliptin treatment increased active GLP-1 by 3-fold, total GLP-1 by 1.5-fold, and total GIP by 2-fold, respectively. Pro^3^, Ex-9, or the combination of both Pro^3^ and Ex-9 exerted no effects on the total and active GLP-1 concentrations or total GIP concentration in plasma. Vildagliptin treatment exerted no effect on glucose tolerance after oral administration of glucose in the nondiabetic *Apoe* ^−/−^ mice (Figure (Fig.) 1).

**Table 1:** Characteristics and laboratory data of nondiabetic *Apoe* ^−/−^ mice.

	VehicleVilda (−)(n = 11)	VehicleVilda (+)(n = 25)	Pro^3^Vilda (+)(n = 7)	Ex-9Vilda (+)(n = 7)	Pro^3^+Ex-9Vilda (+)(n = 8)	Pro^3^+Ex-9Vilda (−)(n = 6)
Food Intake (g/day)	3.7±0.3	3.5±0.1	3.5±0.2	3.6±0.1	3.5±0.1	3.6±0.2
Water Intake (ml/day)	3.8±0.3	3.5±0.2	3.4±0.1	3.5±0.1	3.6±0.1	3.8±0.2
Vildagliptin Intake (mg/kg/day)	0	3.3±0.1	3.2±0.1	3.3±0.1	3.4±0.1	0
Body Weight (g)	31±0.6	32±0.3	32±1	31±1.2	32±0.9	31±0.7
SBP (mm Hg)	99±8	99±3	96±3	100±3	96±5	100±3
Pulse Rate (beat/min)	650±9	618±15	631±12	617±11	652±15	625±10
Total Cholesterol (mmol/l)	11.1±0.67	9.40±0.31	10.3±0.80	9.09±0.78	10.6±1.04	10.4±1.24
HDL Cholesterol (mmol/l)	0.49±0.05	0.36±0.03	0.36±0.05	0.26±0.05	0.34±0.05	0.26±0.05
Non-HDL Cholesterol (mmol/l)	10.6±0.65	9.04±0.31	9.97±0.80	8.81±0.73	10.2±1.01	10.2±1.19
Triglyceride (mmol/l)	1.39±0.19	1.07±0.07	0.91±0.10	0.81±0.20	0.76±0.11	0.82±0.08
NEFA (mmol/l)	1.03±0.05	0.9±0.04	0.99±0.05	1.08±0.08	1.1±0.13	1.3±0.17
Glucose (mmol/l)	8.9±0.6	8.8±0.4	8.0±0.4	7.8±0.8	9.0±1.0	8.8±1.1
Insulin (pmol/l)	88±17	90±8	95±12	113±15	111±19	90±16
Active GLP-1 (pmol/l)	1.66±0.3	5.19±0.6 ^a^	5.49±2.9 ^b^	5.32±1.0 ^b^	5.59±2.5 ^b^	1.87±0.5
Total GLP-1 (pmol/l)	6.5±0.3	10.0±0.5 ^a^	11.5±2.6 ^b^	8.5±0.3 ^b^	10.0±2.1 ^b^	NA
Total GIP (pmol/l)	17±2.2	32±3.7 ^b^	NA	29±3 ^b^	NA	NA
HbA1c (%)	4.4±0.1	4.2±0.1	NA	NA	NA	NA

Mean ± SEM. ^a^ *P*<0.01, ^b^ *P*<0.05 vs. control, NEFA  =  non esterified fatty acids, NA  =  not assayed.

Table 2 shows characteristics and laboratory data from the 5 groups of diabetic *Apoe* ^−/−^ mice treated with vehicle, vildagliptin, vildagliptin with Pro^3^, vildagliptin with Ex-9, and vildagliptin with Pro^3^ and Ex-9. These diabetic mice exhibited the classical features of STZ-induced diabetes: *i.e.*; severe hyperglycemia, hyperphagia, low body weight gain, and low insulin concentration. They also had elevated levels of total and non-HDL cholesterols and NEFA as well as glucose compared with nondiabetic counterparts. There were virtually no differences among the diabetic subgroups in food intake, body weight, SBP, or plasma concentrations of total cholesterol, HDL cholesterol, or triglyceride. As a result of severe hyperglycemia, the STZ-induced diabetic mice lost 20% of their body weight and consumed 1.6 times more drinking water than nondiabetic mice. This effectively doubled the vildagliptin intake in the diabetic mice compared with the intake of the nondiabetic mice. Vildagliptin had no effects on plasma glucose or insulin levels in the 6-h fasting state, but it significantly decreased HbA1c from 8.7 to 7.1%. This HbA1c reduction was abolished by Ex-9 infusion, but not by Pro^3^ infusion. It thus appeared that the HbA1c-lowering effect of vildagliptin was solely due to the action of GLP-1. Plasma levels of active GLP-1, total GLP-1, and total GIP were 2–4 fold higher in diabetic mice than in nondiabetic mice. Active GLP-1 concentrations were ∼3-fold higher in all vildagliptin-treated groups compared with the non-treated mice. Pro^3^, Ex-9, and both had no effects on active GLP-1 concentrations. Vildagliptin increased total GLP-1 by 3-fold and GIP by 2-fold, respectively. Pro^3^, Ex-9, and both had no effects on total GLP-1 levels, and Ex-9 had no effect on total GIP levels.

**Table 2:** Characteristics and laboratory data of diabetic *Apoe* ^−/−^ mice.

	VehicleVilda (−)(n = 6)	VehicleVilda (+)(n = 9)	Pro^3^Vilda (+)(n = 7)	Ex-9Vilda (+)(n = 8)	Pro^3^+Ex-9Vilda (+)(n = 6)
Food Intake (g/day)	4.4±0.3	4.3±0.3	4.1±0.2	4.1±0.3	4.2±0.2
Water Intake (ml/day)	6.1±0.2	5.8±0.1	5.9±0.2	5.8±0.1	6.0±0.1
Vildagliptin Intake (mg/kg/day)	0	6.9±0.1	7.3±0.1	7.2±0.1	7.5±0.1
Body Weight (g)	23.7±1.1	25.1±1.1	24.4±1.1	24.2±1.6	24.0±1.2
SBP (mm Hg)	102±6	101±5	98±4	99±8	99±8
Pulse Rate (beat/min)	620±18	604±32	611±22	615±19	621±30
Total Cholesterol (mmol/l)	17.3±1.74	15.9±2.51	16.8±0.75	16.3±2.46	16.1±0.78
HDL Cholesterol (mmol/l)	0.23±0.03	0.26±0.05	0.26±0.05	0.21±0.05	0.26±0.03
Non-HDL Cholesterol (mmol/l)	17.0±1.76	15.6±2.51	16.1±0.75	16.1±2.46	15.8±0.78
Triglyceride (mmol/l)	0.44±0.14	0.34±0.07	0.46±0.07	0.44±0.10	0.43±0.09
NEFA (mmol/l)	1.42±0.20	1.10±0.13	1.37±0.15	1.02±0.06	1.31±0.29
Glucose (mmol/l)	19.0±0.7	17.9±0.6	20.1±1.7	18.4±2.1	18.6±2.2
Insulin (pmol/l)	38±12	53±11	48±10	67±21	60±10
Active GLP-1 (pmol/l)	3.48±1.20	9.40±1.89 ^a^	10.10±1.98 ^a^	10.05±2.08 ^a^	9.97±1.27 ^a^
Total GLP-1 (pmol/l)	18.4±5.9	53.5±14.6 ^a^	56.1±16.2 ^a^	60.9±13.5 ^a^	39.7±13.7 ^a^
Total GIP (pmol/l)	73.9±6.0	145.2±13.9 ^a^	NA	146.1±27.7 ^a^	NA
HbA1c (%)	8.7±0.4	7.1±0.3 ^b^	7.1±0.3 ^b^	8.9±0.5	8.6±0.4

Mean ± SEM. ^a^ *P*<0.05, ^b^ *P*<0.01 vs. control. NEFA  =  non esterified fatty acids, NA  =  not assayed.

### Atherosclerotic lesions

**Figure 2:**
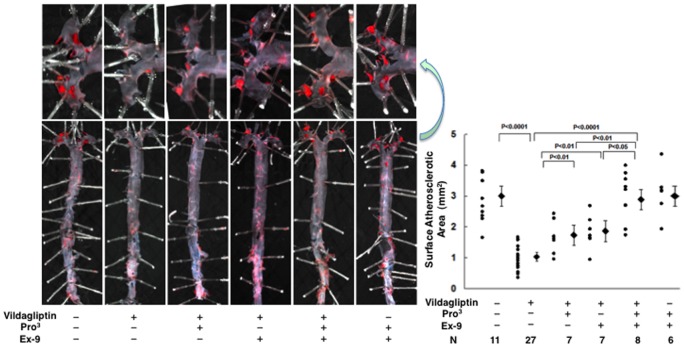
Surface atherosclerotic lesions of the aorta in 21-week-old nondiabetic *Apoe* ^−**/**−^ mice treated without/with vildagliptin infused with incretin receptor blockers. During the 4-week period of vildagliptin administration, the *Apoe* ^−/−^ mice (17-weeks-old at the outset) were respectively infused with saline (vehicle), GLP-1 receptor blocker (Ex-9), the GIPR blocker (Pro^3^), and Ex-9+Pro^3^ by osmotic mini-pump. The entire aorta was stained with Oil Red O.

**Figure 3:**
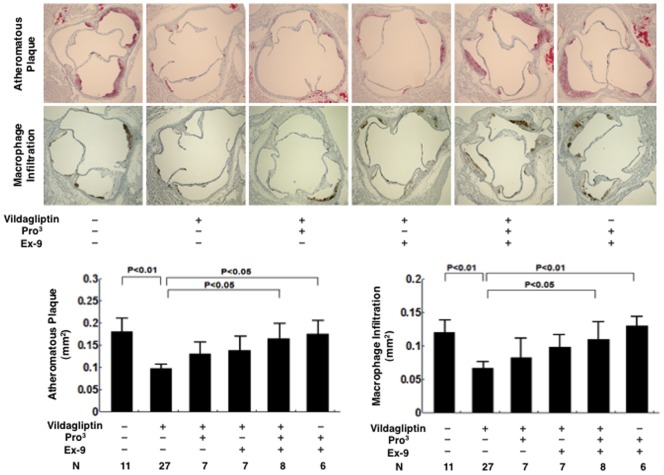
The atherosclerotic area and the macrophage infiltration area in the aortic root in 21-week-old nondiabetic *Apoe* ^−**/**−^ mice treated without/with vildagliptin infused with incretin receptor blockers. The cross-sections of the aortic root were stained with Oil Red O. The area of macrophage infiltration was stained with MOMA-2.

Atherosclerotic lesions were obviously developed in 21-week-old *Apoe* ^−/−^ mice (Figs. 2 and 3). Oral vildagliptin reduced the surface areas of the atherosclerotic lesions by 66% (*P*<0.0001; Fig. 2), reduced the atheromatous plaque size by 46% (*P*<0.01; Fig. 3), and reduced macrophage infiltration by 45% (*P*<0.01; Fig. 3), in the aortic root. Infusion with Pro^3^ and infusion with Ex-9 exhibited comparable effects in partially attenuating the anti-atherosclerotic action of vildagliptin (Figs. 2 and 3). The combination of Pro^3^ and Ex-9 abolished the anti-atherosclerotic action of vildagliptin altogether (Figs. 2 and 3). Infusions with Pro^3^ and Ex-9 to the vehicle controls had no effect on the development of atherosclerosis (Figs. 2 and 3).

**Figure 4:**
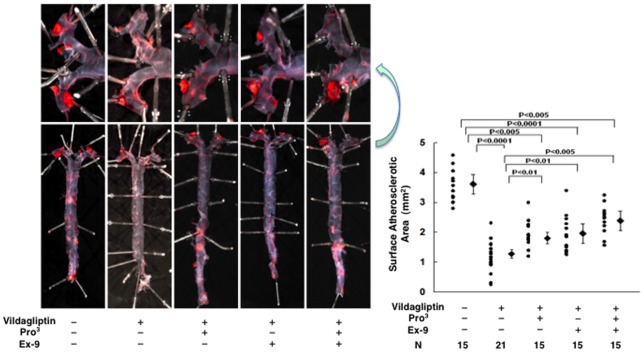
Surface atherosclerotic lesions of the aorta in 21-week-old diabetic *Apoe* ^−**/**−^ mice treated without/with vildagliptin infused with incretin receptor blockers. Diabetes was induced by multiple peritoneal injections of STZ (50 mg/kg/day) for 5 consecutive days. During the 4-week period of vildagliptin administration, the diabetic *Apoe* ^−/−^ mice were respectively infused with saline (vehicle), GLP-1 receptor blocker (Ex-9), the GIPR blocker (Pro^3^), and Ex-9+Pro^3^ for 4 weeks by osmotic mini-pump.

**Figure 5:**
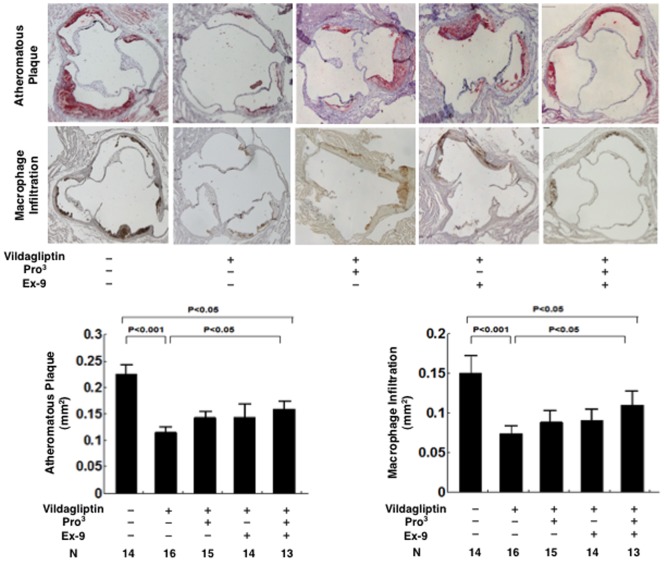
The atherosclerotic area and the macrophage infiltration area in the aortic root in 21-week-old diabetic *Apoe* ^−**/**−^ mice treated without/with vildagliptin infused with incretin receptor blockers. The cross-sections of the aortic root were stained with Oil Red O. The area of macrophage infiltration was stained with MOMA-2.

Severe atherosclerotic lesions developed in the diabetic *Apoe* ^−/−^ mice (Figs. 4 and 5). Vildagliptin reduced the surface areas of the atherosclerotic lesions by 64% (*P*<0.0001; Fig. 4), reduced the atheromatous plaque size by 49% (*P*<0.001; Fig. 5), and reduced macrophage infiltration by 50% (*P*<0.001; Fig. 5) in the aortic root. The suppressive effects of vildagliptin were partially attenuated by the infusions with Pro^3^ or Ex-9 (Figs. 4 and 5). The combination of Pro^3^ and Ex-9 significantly attenuated the suppressive effect of vildagliptin on the surface area of atherosclerosis, atheromatous plaque size, and macrophage infiltration (Figs. 4 and 5). In contrast to the finding in nondiabetic mice, however, the dual receptor blocker did not completely abolish the vildagliptin-induced suppression of the atherosclerotic lesions (Figs. 4 and 5).

### Foam cell formation in exudate peritoneal macrophages from *Apoe* ^−/−^ mice

**Figure 6:**
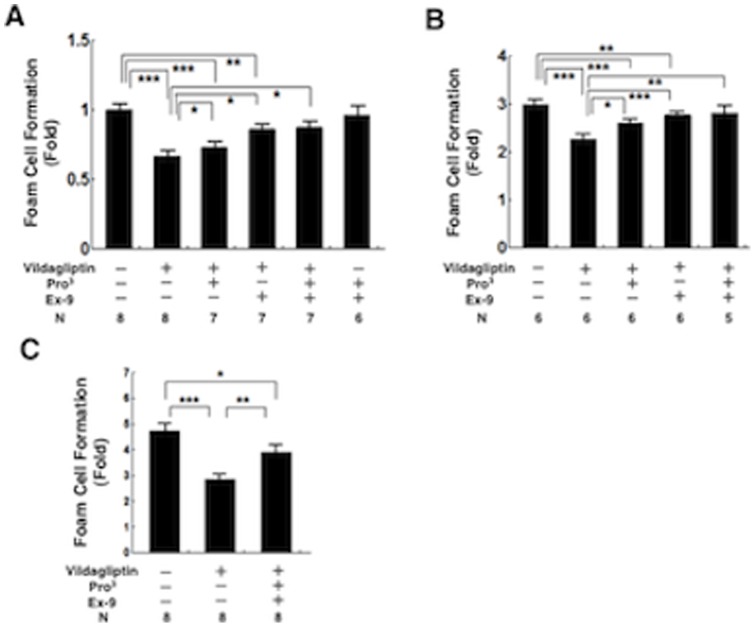
Foam cell formation in exudate peritoneal macrophages. Exudate peritoneal cells were isolated from the treated nondiabetic *Apoe* ^−/−^ mice (a) or diabetic *Apoe* ^−/−^ mice (b) at 21 weeks of age, or *db/db* diabetic mice (c) at the age of 13 weeks, 4 days after an intraperitoneal injection of thioglycolate. Adherent macrophages were incubated for 18 hours with the RPMI-1640 medium containing 10 μg/ml oxLDL in the presence of 0.1 mmol/l [^3^H]oleate conjugated with bovine serum albumin. Cellular lipids were extracted and the radioactivity of the cholesterol [^3^H]oleate was determined by thin-layer chromatography. *P<0.05, **P<0.01, ***P<0.001.

As shown in Fig. 6A, oxLDL-induced cholesterol ester accumulation (foam cell formation) was significantly suppressed in macrophages from vildagliptin-treated *Apoe* ^−/−^ mice vs. vehicle controls (40%, *P*<0.001). The suppressive effects of vildagliptin on foam cell formation *ex vivo* were significantly attenuated by the infusions with Ex-9, Pro^3^, and the combination of Ex-9 and Pro^3^ (Fig. 6A). Infusions with Ex-9 and Pro^3^ to vehicle controls had no effect on foam cell formation (Fig. 6A). OxLDL-induced foam cell formation was 3-fold higher in macrophages obtained from diabetic *Apoe* ^−/−^ mice than in macrophages from nondiabetic *Apoe* ^−/−^ mice (Fig. 6B). Foam cell formation was significantly suppressed in macrophages from vildagliptin-treated diabetic *Apoe* ^−/−^ mice vs. vehicle controls (24%, *P*<0.001). The suppressive effect of vildagliptin on foam cell formation *ex vivo* was almost completely eliminated by the infusions with Pro^3^, Ex-9, and the combination of Pro^3^ and Ex-9 (Fig. 6B).

### Foam cell formation in exudate peritoneal macrophages from *db/db* mice

Table 3 shows characteristics and laboratory data from the *db/db* diabetic mice treated with vehicle, with vildagliptin, and with vildagliptin combined with Pro^3^ and Ex-9. There were virtually no differences among the groups in food intake, water intake, body weight, SBP, pulse rate, or the plasma concentrations of total cholesterol, HDL cholesterol, or glucose (6-h fast). Vildagliptin treatment increased insulin by 51%, increased active GLP-1 by 315%, and increased total GIP by 23%. Vildagliptin significantly reduced HbA1c levels, and Pro^3^ and Ex-9 attenuated the hypoglycemic effect of vildagliptin. OxLDL-induced foam cell formation was ∼5-fold higher in macrophages obtained from diabetic *db/db* mice than in macrophages from nondiabetic *Apoe* ^−/−^ mice. Foam cell formation was significantly suppressed in macrophages from vildagliptin-treated *db/db* mice vs. vehicle controls (40%, *P*<0.001; Fig. 6C). The suppressive effects of vildagliptin on foam cell formation *ex vivo* were remarkably attenuated by the infusions with the combination of Pro^3^ and Ex-9 (Fig. 6C).

**Table 3:** Characteristics and laboratory data of *db/db* diabetic mice.

	VehicleVilda (−)(n = 11)	VehicleVilda (+)(n = 11)	Pro^3^+Ex-9Vilda (+)(n = 11)
Food Intake (g/day)	4.4±0.3	4.3±0.3	4.2±0.2
Water Intake (ml/day)	6.0±0.2	6.1±0.2	5.8±0.1
Vildagliptin Intake (mg/kg/day)	0	3.8±0.1	3.8±0.1
Body Weight (g)	46.1±0.7	46.8±0.5	46.4±0.6
SBP (mm Hg)	111±8	107±6	112±10
Pulse Rate (beat/min)	580±21	564±42	530±30
Total Cholesterol (mmol/l)	3.29±0.29	3.19±0.14	3.16±0.20
HDL Cholesterol (mmol/l)	0.23±0.03	0.26±0.05	0.26±0.03
Non-HDL Cholesterol (mmol/l)	3.06±0.29	2.93±0.13	2.90±0.20
Glucose (mmol/l)	18.1±1.8	16.5±1.6	17.3±2.2
Insulin (pmol/l)	777±86	1175±52 ^a^	1019±52 b
Active GLP-1 (pmol/l)	3.73±0.8	15.5±2.3 ^a^	14.1±1.6 b
Total GLP-1 (pmol/l)	52.7±5.7	54.6±6.2	53.0±5.0
Total GIP (pmol/l)	53.0±6.0	65.0±4.0 ^b^	NA
HbA1c (%)	7.6±0.3	6.6±0.2 ^b^	NA

Mean ± SEM. ^a^ *P*<0.01, ^b^ *P*<0.05 vs. control. NA  =  not assayed.

### Incretin receptor gene expression

**Figure 7:**
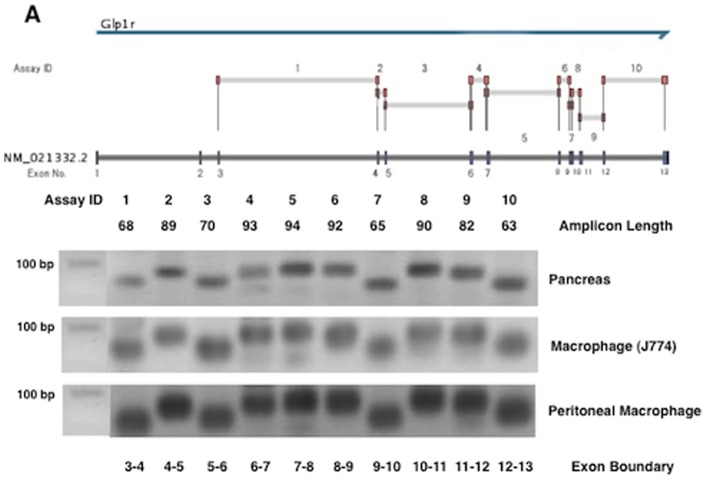
GLP-R gene expression. GLP-1R mRNA levels in the pancreas and the exudate peritoneal macrophages obtained from *Apoe* ^−/−^ mice, and J774A1 mouse macrophages were determined by real-time RT-PCR with 10 primers encoding different exons of the GLP-R shown in Supplementary data. The amplification products visualized by gel electrophoresis had the expected lengths (bp).

**Figure 8:**
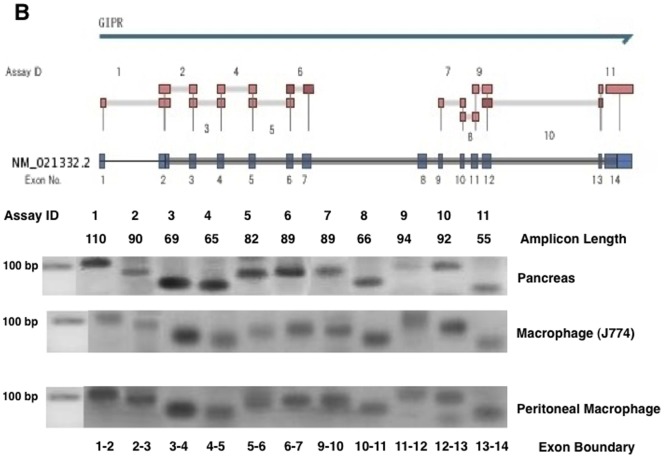
GIPR gene expression. GIPR mRNA levels in the pancreas and the exudate peritoneal macrophages obtained from *Apoe* ^−/−^ mice, and J774A1 mouse macrophages were determined by real-time RT-PCR with 11 primers encoding different exons of the GIPR shown in Supplementary data. The amplification products visualized by gel electrophoresis had the expected lengths (bp).

**Figure 9:**
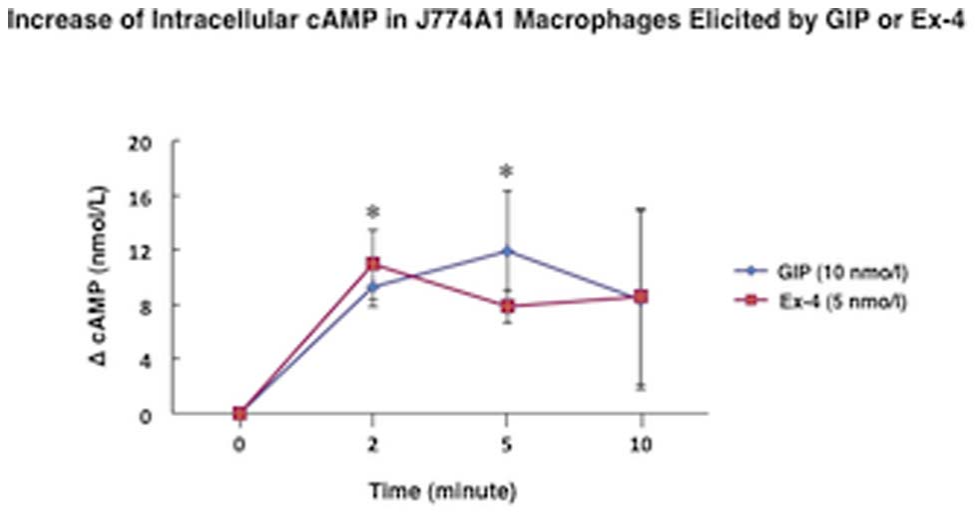
Increase of intracellular cAMP in J774A1 macrophages elicited by excendin-4 or GIP. *P<0.05 vs. 0 minute.

To detect GLP-1R gene expression, we used 10 primers that encoded virtually all of the exons of mouse GLP-1R. As shown in Fig. 7, all of the GLP-1R mRNA corresponding to the exon boundary was detected in a mouse macrophage cell line (J774A1) and in peritoneal macrophages obtained from *Apoe* ^−/−^ mice. Each amplified product of the GLP-1R gene in the mouse macrophages migrated to the same expected position in the pancreas, a standard GLP-1R-expressing organ. Likewise, GIPR gene products corresponding to each exon boundary (1–14 exons) were detected in J774A1 and the peritoneal macrophages. Each mRNA band encoded by the GIPR gene migrated to the same amplicon length of the pancreas (Fig. 8). GLP-1R gene expression, however, was far less abundant in both types of macrophages than in the vasculature, pancreas, or brain (Table 4). When the gene expression of GLP1-R in the vasculature was 1.000, its expression in the peritoneal macrophages was less than 0.014 (Table 4). The gene expression of GIPR was very weak in the peritoneal macrophages, like that of GLP-1R, but stronger in J774A1 (Table 5). We determined the effect of incretins on cAMP generation in the J774A1 cells. GIP and the GLP-1R agonist exendin-4 (Ex-4) both elicited significant cAMP generation in the mouse macrophages (Fig. 9).

**Table 4:** Expressions of GLP-1R in various mouse tissues or cells.

GLP-1R mRNA	1	2	3	4	5	6	7	8	9	10
Vasculature	1.001	1.001	1.002	0.998	0.999	1.001	1.000	1.000	0.999	0.998
Macrophage	0.014	0.0004	0.004	0.007	0.005	0.006	0.003	0.005	0.004	0.003
J774A.1	0.014	0.002	0.007	0.005	0.002	0.003	0.370	0.002	0.004	0.004
Adipose Tissue	0.104	0.004	0.032	0.072	0.056	0.224	0.045	0.085	0.041	0.007
Pancreas	160.8	84.75	29.55	20.85	41.07	29.99	36.91	46.22	36.71	33.45
Brain	18.80	22.35	19.23	12.75	9.067	13.54	44.14	15.03	9.36	6.96

**Table 5:** Expressions of GIPR in various mouse tissues or cells.

GIPR mRNA	1	2	3	4	5	6	7	8	9	10	11
Vasculature	1.000	1.001	0.998	1.003	0.997	1.002	1.002	1.001	1.002	1.002	1.002
Macrophage	0.015	0.004	0.237	0.005	0.051	0.274	0.003	0.005	0.001	0.135	0.003
J774A.1	0.454	0.416	3.080	1.158	2.745	2.688	0.481	0.435	0.836	1.859	0.018
Adipose Tissue	3.213	3.725	2.528	4.145	25.95	2.799	4.110	4.747	7.294	2.795	5.101
Pancreas	7.688	5.379	94.91	6.864	49.42	82.50	8.667	9.705	9.140	7.036	3.279
Brain	6.087	13.63	3.567	10.03	101.3	7.193	13.65	11.74	22.63	11.81	7.578

Relative gene expressions of GLP-1R and GIP in various mouse tissues or cells determined by real-time PCR using 10 and 11 primers when the gene expression of the vasculature (aorta) is settled as 1.000.

### DPP-4/CD26 gene expression in the peritoneal macrophages obtained from nondiabetic and diabetic wild-type mice or *Apoe* ^−/−^ mice

**Figure 10:**
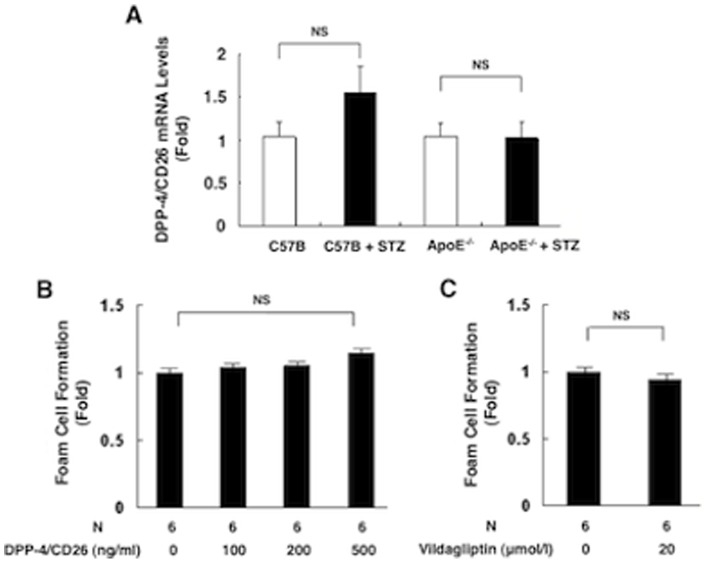
DPP-4/CD26 gene expression in macrophages and the *in vitro* effect of DPP-4/CD26 on foam cell formation in macrophages. DPP-4/CD26 mRNA levels were measured in peritoneal macrophages obtained from nondiabetic mice and STZ-induced diabetic wild-type (C57/BL) or *Apoe* ^−/−^ mice (A). Neither DPP-4/CD26 (100–500 ng/ml) (B) nor vildagliptin (20 μmol/l) affected the oxLDL-induced foam cell formation in peritoneal macrophages obtained from nondiabetic *Apoe* ^−/−^ mice (C).

DPP-4/CD26 mRNA levels in the peritoneal macrophages were comparable between nondiabetic mice and STZ-induced diabetic mice, irrespective of wild-type or *Apoe* ^−/−^ mice (Fig. 10A).

### Effect of DPP-4/CD26 on macrophage foam cell formation *in vitro*

DPP-4/CD26 (100–500 ng/ml) had no effect on oxLDL-induced macrophage foam cell formation in peritoneal macrophages obtained from nondiabetic *Apoe* ^−/−^ mice (Fig. 10B). A high concentration of vildagliptin (20 μmol/l) had no effect on oxLDL-induced foam cell formation in peritoneal macrophages obtained from nondiabetic *Apoe* ^−/−^ mice (Fig. 10C).

## Discussion

The present study confirmed our previous observation [8] by showing once again that a DPP-4 inhibitor significantly suppressed the development of atherosclerosis in *Apoe* ^−/−^ mice fed an atherogenic diet. Yet the mechanism by which DPP-4 inhibitors suppress the development of atherosclerosis has yet to be fully elucidated. While GLP-1 and GIP represent important targets for DPP4 activity, it remains uncertain whether additional substrates, such as stromal cell-derived factor-1α (SDF-1α), are important for the anti-atherogenic actions of DPP-4 inhibitors remains uncertain. At least, our preliminary study showed that SDF-1α did not significantly affect macrophage foam cell formation (data not shown). Other studies have suggested that DPP-4/CD26 is an atherogenic molecule itself and that DPP-4 inhibitors exert anti-atherogenic effects by inactivating DPP-4 [5], [6], [9], [10]. The present study was the first to examine whether the anti-atherosclerotic action of DPP-4 inhibitor is mainly attributable to the action of incretins or to other mechanisms.

Ex-9 or Pro^3^ partially attenuated the vildagliptin-induced suppression of atherosclerosis, whereas the combination of Ex-9 and Pro^3^ completely abolished the vildagliptin-mediated suppression of atherosclerosis in nondiabetic *Apoe* ^−/−^ mice. This finding suggests that the actions of both incretins may protect against the development of atherosclerosis, while a single incretin action may be insufficient to exert a full anti-atherosclerotic. Our results resemble an earlier study in which glucose intolerance turned out to be more severe in double incretin receptor knockout mice than in single incretin receptor knockout mice [20]. Hansotia *et al*. [21] found that DPP-4 inhibitor improved glycemic excursion in association with increased levels of circulating insulin in wild-type and single incretin receptor knockout mice, whereas DPP-4 inhibitor had no significant effect on glucose or insulin secretion in double incretin receptor knockout mice. This study suggests that the glucose-lowering properties of DPP-4 inhibitors are mediated exclusively *via* the enhanced action of the two incretins.

The insulinotropic action of GIP virtually disappears in diabetes because of the severe down-regulation of GIPR in the pancreatic islets [11], [12]. Thus, the results obtained from nondiabetic animals cannot easily ensure the results obtained from diabetic animals with abnormal GIPR. We recently reported that GIP infusion remarkably suppressed the development of atherosclerosis in STZ-induced diabetic *Apoe* ^−/−^ mice, and GIP suppressed macrophage foam cell formation obtained from the diabetic mice though GIPR was mildly down-regulated in the macrophage [14]. The high plasma concentration of GIP obtained through GIP infusion may be sufficient to exert an anti-atherogenic effect even when GIPR expression is mildly reduced. Yet it remained unknown whether the physiological increase of GIP by DPP-4 inhibition exerted an anti-atherogenic effect similar to that exerted by GIP infusion in diabetic animals. We have now found that vildagliptin remarkably suppresses the development of atherosclerosis while inhibiting macrophage foam cell formation by mechanism similar to those observed in nondiabetic mice. Consistent with our observation, Ta *et al*. [6] reported that alogliptin, another DPP-4 inhibitor, substantially suppressed atherosclerotic lesions in STZ-induced diabetic *Apoe* ^−/−^ mice. We measured total GIP but not active GIP, as no active GIP assay kit is commercially available. Nevertheless, diabetic mice had 4-fold higher total GIP than nondiabetic mice, and vildagliptin doubled the total GIP in diabetic mice, pushing the active GIP concentration to a level at least 8-fold higher than the levels in nondiabetic and non-vildagliptin-treated mice. GIP at this high concentration might be sufficient to overcome the mild down-regulation of GIPR in macrophages and exert anti-atherogenic effects. Vildagliptin significantly decreased HbA1c levels, and this hypoglycemic effect was attenuated by Ex-9 but not by Pro^3^. The glucose-lowering action of vildagliptin *via* the increase of active GLP-1 is very likely to be involved in mechanisms behind the vildagliptin-induced suppression of atherosclerosis. On the other hand, the action of Pro^3^ in attenuating anti-atherogenic action without affecting HbA1c suggests that the enhancement of active GIP by DPP-4 inhibition suppresses atherosclerosis through direct action.

Drucker's group [22] recently reported that a GLP-1 analog, taspoglutide, was ineffective as a suppressor of atherosclerosis in STZ-induced diabetic *Apoe* ^−/−^ mice fed a fat-rich diet. Further, they were unable to detect GLP-1R gene expression in peritoneal macrophages obtained from C57/BL mice and *Apoe* ^−/−^ mice. Yet several previous studies have demonstrated that incretin signals can enter the macrophages, by confirming that GLP-1 stimulates the cAMP/PKA pathway and exerts an anti-inflammatory property in macrophages obtained from mice [7], [23], . Since Ex-9 and Pro^3^ are competitive antagonists for the classical incretin receptors, the present data imply that anti-atherogenic action of incretins are mediated by the incretin receptors. We identified the expression of GLP-1R and GIPR in murine macrophages using multiple primers encoding different exons covering the whole incretin receptor gene. We also confirmed that GLP-1R agonist (Ex-4) or GIP elicited cAMP generation in J774A1 mouse macrophages. Yet the gene expressions of GLP-1R and GIPR were far less abundant in the macrophages than in those in the pancreas or vasculature. It thus remains uncertain whether the anti-atherogenic effect of incretins induced by vildagliptin were exerted *via* incretin receptors in the macrophages. We previously observed an extreme down-regulation of the incretin receptor gene expression in monocytes when the monocytes differentiated into macrophages [4]. The incretins might exert their anti-atherogenic effect only when the incretin receptors are abundantly expressed in the monocytes. Further studies will be required to elucidate this possibility.

It has recently been hypothesized that DPP-4/CD26 is a pro-inflammatory and pro-atherogenic molecule that may promote atherogenic processes [3], [10]. When we examined the *in vitro* effects of DPP-4/CD26 and its inhibitor on macrophage foam cell formation, an essential process of early atherosclerosis, DPP-4/CD26 showed no direct regulatory action on macrophage foam cell formation. This finding reinforces our conclusion that the anti-atherosclerotic effect of a DPP-4 inhibitor mainly stems from the action of incretin. In contrast to what we found in nondiabetic mice, the combination of GLP-1R and GIPR blockers only partially attenuated the anti-atherogenic effect of vildagliptin in diabetic mice. The increased drinking water intake associated with hyperphagia doubled the vildagliptin intake in diabetic *Apoe* ^−/−^ mice compared to their nondiabetic counterparts. Thus, the infused incretin receptor blockers in diabetic mice may be insufficient to antagonize the increase in incretins brought about by the double vildagliptin dose. Alternatively, the incomplete prevention of atherosclerotic lesions by the dual incretin receptor antagonists in diabetic mice implies that vildagliptin confers a partial anti-atherogenic effect beyond that conferred by incretins in diabetes, a disease with aggravated vascular inflammation.

The present study has some potential limitations. First, the diabetic mice received an almost 2-fold higher dose of vidagliptin than the nondiabetic mice. This dose differential made it difficult to simply compare the incretin-dependent and incretin-independent actions of vildagliptin on anti-atherogenesis between the diabetic and nondiabetic mice. Second, we neglected to investigate the direct anti-atherogenic effect of incretins on murine monocytes that would have expressed incretin receptors more abundantly than macrophages. Third, we only partially evaluated the incretin-independent effect of vildagliptin on the suppression of atherosclerosis in the diabetic mice. Future work is warranted to confirm these findings.

## Conclusions

Vildagliptin exhibited a substantial anti-atherosclerotic effect by inhibiting macrophage foam cell formation in both nondiabetic and diabetic mice, mainly *via* the actions of GLP-1 and GIP. There is still a possibility, however, that the anti-atherogenic effect of DPP-4 inhibitors is partly independent of the action of the incretins in diabetic mice.
